# Nano-sized Al_2_O_3_ reduces acute toxic effects of thiacloprid on the non-biting midge *Chironomus riparius*

**DOI:** 10.1371/journal.pone.0176356

**Published:** 2017-05-02

**Authors:** Carla S. Lorenz, Anna-J. Wicht, Leyla Guluzada, Leilei Luo, Leonie Jäger, Barbara Crone, Uwe Karst, Rita Triebskorn, Yucang Liang, Reiner Anwander, Stefan B. Haderlein, Carolin Huhn, Heinz-R. Köhler

**Affiliations:** 1 Institute of Evolution and Ecology, Animal Physiological Ecology, University of Tübingen, Tübingen, Germany; 2 Institute of Physical and Theoretical Chemistry, University of Tübingen, Tübingen, Germany; 3 Center for Applied Geosciences, Environmental Mineralogy and Chemistry, University of Tübingen, Tübingen, Germany; 4 Institute of Inorganic Chemistry, University of Tübingen, Tübingen, Germany; 5 Institute of Inorganic and Analytical Chemistry, University of Münster, Münster, Germany; 6 Steinbeis Transfer-Center for Ecotoxicology and Ecophysiology, Rottenburg, Germany; VIT University, INDIA

## Abstract

This study focuses on interactions between nanoparticles and a pesticide. The aim was to investigate how nano-sized aluminum oxide (410 nm) can alter the toxic effects of thiacloprid, even if no sorption between particles and the insecticide takes place. Thus, our study investigated a rather unexplored interaction. We conducted our research with larvae of *Chironomus riparius* and used thiacloprid as test substance as its toxicity to *C*. *riparius* is well described. The used nano-Al_2_O_3_ particles where chosen due to their suitable properties. For testing the acute effects of the interaction, we exposed larvae to thiacloprid (0.5, 1.0, 2.0, and 5.0 μg/L) and nano-Al_2_O_3_ (300 and 1000 mg/L), either solely or in binary mixtures. While thiacloprid resulted in elevated mortality, nano-Al_2_O_3_ solely did not exert any effects. Moreover, we observed an aggregation of nano-Al_2_O_3_ within the lumen of the intestinal tract of the larvae. Further results showed a significantly reduced mortality of fourth instar larvae when they were exposed to mixtures of nanoparticles and the pesticide, compared to thiacloprid alone. With increasing nano-Al_2_O_3_ concentration, this effect became gradually stronger. Additionally, chemical analyses of internal thiacloprid concentrations implicate reduced uptake of thiacloprid in animals exposed to mixtures. However, as larvae exposed to thiacloprid concentrations > 0.5 μg/L showed severe convulsions, independent of the presence or concentration of nano-Al_2_O_3,_ we assume that nano-Al_2_O_3_ leads to a delay of mortality and does not entirely prevent it. As sorption measurements on pristine or defecated nano-Al_2_O_3_ did not reveal any sorptive interaction with thiacloprid, we can exclude sorption-based reduction of thiacloprid bioavailability as a mechanism behind our results. Even though we used test substances which might not co-occur in the environment in the tested concentrations, our study gives evidence for an interaction besides adsorption, which is important to generally understand how nanoparticles might affect biota.

## 1. Introduction

From an environmental point of view nanoparticles (i.e. particles with a size of 1–100 nm) are no particular novelty raised in the centuries of industrialization, as they are formed naturally, e.g. by volcanic activities or forest fires [1–4]. However, nowadays, nanoparticles are specifically synthesized to find their application in electronics, medicine, human care products, and many other sectors [2, 5]. Although engineered nanoparticles (ENPs) are frequently used, and their release, e.g. via wastewater, is very likely, little is known about their fate and effects in the environment [1, 6–8]. Due to these reasons, ENPs have the potential to pose a novel threat to the environment and, consequently, come to focus in ecotoxicology [2, 7, 9]. Many factors, like their abundance in nature, transport within biota and their environment, and their possible persistence, all confounded by their unique properties, e.g. a large reactive surface area per volume, need to be considered to estimate potential hazards of nanoparticles [2, 5, 8, 10]. Consequently, the risk assessment of such particles is highly complex. Moreover, nanoparticles can interact with various substances like pollutants or nutrients, a phenomenon, which is also relevant for risk assessment [10–12]. For example, the adsorption of pollutants by nanoparticles can lead, amongst others, to reduced bioavailability of the contaminant and, consequently, reduced toxicity [2, 11]. Particularly this kind of interaction, adsorption, is in the focus of most studies, but very little attention has been paid to effects based on the presence of chemicals and non-adsorbing nanoparticles. Therefore, potential effects of this kind of interaction are more or less unknown yet. To fill this gap, we aimed at investigating whether nano-sized Al_2_O_3_ particles interfere with the toxicity of the neonicotinoid insecticide thiacloprid, even though no sorptive interaction between both substances was measured. A co-occurrence of both substances in the environment is theoretically possible, even though no data are yet available. We have chosen these substances, because the effects of thiacloprid have already been described for our study organism [13], the non-biting midge *Chironomus riparius*, which facilitates the interpretation of our results. Nano-Al_2_O_3_ particles were used, as they did not show any adsorption of thiacloprid in sorption experiments. Moreover, as nano-Al_2_O_3_ particles will settle to the substratum, the sediment-dwelling and detritus feeding larvae of *C*. *riparius* [14] will be directly exposed to these particles, as has been predicted for benthic organisms in general [7].

To investigate the effects of a non-sorptive interaction, we conducted acute toxicity tests with fourth instar larvae of *C*. *riparius*, where we assessed the effects of both test substances–solely and in combination–on the mortality rate and the larvae’s behavior. Furthermore, these tests were accompanied by chemical analyses of internal thiacloprid concentrations.

In summary, our main questions addressed in this study were the following:

1. Do thiacloprid and nano-Al_2_O_3_ interact with one another?
2. Does nano-Al_2_O_3_ alter thiacloprid uptake and toxicity in a non-target insect?

## 2. Material and methods

### 2.1. Ethics statement

The used model organism of this study (*Chironomus riparius*) is a nonregulated species and no ethical approval was required for conducting the experiments.

### 2.2. Cultivation of *Chironomus riparius*

*Chironomus riparius* belongs to the family Chironomidae (order Diptera), which is one of the most abundant insect families in freshwater ecosystems and thus represents an important part of food webs [14]. Moreover, it is a well-established model organism in ecotoxicology [13, 15, 16]. The cultivation was maintained in our laboratory at the University of Tübingen. A stock culture was established with individuals obtained from Goethe University Frankfurt (Germany). The animals were kept in plastic basins (30 x 55 x 12 cm), containing a bottom layer of fine quartz sand (thickness: 3 cm, particle size: 0.1-0.3 mm), covered by filtered and dechlorinated tap water (filtered by iron and active carbon filters) with continuous, gentle aeration. Larvae were fed with ground TetraMin^®^ fish flakes (Tetra, Germany) every second day, and 50% of the water volume was changed once per week. All basins were covered by breeding cages (55 x 65 x 120 cm; mesh material with a mesh size of 0.5 mm^2^) to allow adult midges to swarm and mate. The whole set-up of five breeding cages was located in a climate chamber with a temperature of 21.0°C ± 0.5°C and a light-dark cycle of 16:8 h.

### 2.3. Nano-Al_2_O_3_ synthesis

The synthesis of the alumina particles was performed according to a slightly modified literature procedure [17]. Aluminum sulfate hexadecahydrate (2.00 g), aluminum nitrate nonahydrate (4.43 g), and urea (9.00 g) were dissolved in 2.0 L of deionized water. The solution was stirred and heated to 98°C, and subsequently kept at this temperature for 1.5 h. The formed hydrous alumina particles were separated by centrifugation, washed three times with deionized water, and dried at 80°C overnight. The alumina particles (S1 Fig) had a mean size of 410 nm (Transmission electron microscopy, JEOL JEM 2010 microscope operated at 160 kV) with a surface area of 9.0 m^2^/g and a zeta potential (deionized water) of +20.4 mV (Malvern Zetasizer Nano-ZS, Malvern Instruments Ltd, UK). Considering the size of these hydrous alumina particles, they, strictly speaking, do not belong to nanoparticles per definition, but are still within the nanoscale range.

### 2.4. Preparation of test solutions

Nano-Al_2_O_3_ and thiacloprid were tested as single substances or mixtures in different concentrations. Since we aimed at investigating aspects of compound interactions as a matter of principle and did not focus on environmentally relevant concentrations, we used rather high concentrations of thiacloprid and nano-Al_2_O_3_ particles to generate possible effects even under acute exposure conditions. All treatments are listed in Table 1.

**Table 1:** Tested substances and combinations.

Treatment	Concentration of test substances		
	Nano-Al_2_O_3_ [mg/L]	Thiacloprid [μg/L] nominal	Thiacloprid [μg/L] measured
**Control**	0	0	0
**Nano-Al_2_O_3_ solely,** **2 concentrations**	300, 1000	0	Not measured
**Thiacloprid solely,** **4 concentrations**	0	0.5, 1.0, 2.0, 5.0	0.4, 0.7, 1.6, 4.0
**Mixtures, Type A,** **4 concentrations**	300	0.5, 1.0, 2.0, 5.0	0.4, 0.8, 1.3, 3.5
**Mixtures, Type B,** **4 concentrations**	1000	0.5, 1.0, 2.0, 5.0	0.2, 0.8, 1.6, 3.7

All test concentrations were prepared with filtered and dechlorinated tap water directly before use. For the preparation of those test solutions that contained thiacloprid, a stock solution of 5 mg/L thiacloprid (analytical standard, Sigma Aldrich, Germany) was prepared with filtered and dechlorinated tap water, and stirred overnight in the dark at 7°C. For tests with nano-Al_2_O_3_ as single substance and for the mixture experiments, the nano-Al_2_O_3_ particles were added to the test solutions and ultrasonicated for 15 min at room temperature.

### 2.5. Acute toxicity test

Prior to all experiments, all test vessels (glass, diameter: 7 cm, height: 6.5 cm) were saturated with the corresponding test solutions overnight. Subsequently, all test vessels were emptied and refilled with 30 g fine quartz sand (particle size: 0.1–0.3 mm) and 100 mL of the corresponding test solution, or filtered and dechlorinated tap water as control. Twelve replicates for each of the fifteen treatments were tested, resulting in the exposure of 60 larvae per treatment. To ascertain the test solutions and the stock culture to have the same temperature (~ 21°C), the test vessels were acclimatized for two hours in a climate chamber. Additionally, the temperatures of the test solutions were measured to guarantee that test animals did not suffer from thermal stress. At the start of the experiments, five fourth instar larvae of *C*. *riparius* were inserted into each test vessel using a blunt glass pipette. Test vessels were placed on a table in a random arrangement and were covered with perforated Parafilm^®^ (Carl Roth GmbH, Germany) to reduce evaporation.

Larvae were exposed for 96 h, and no food was added to prevent possible interactions with the test compounds and to decrease the animals´ oxygen consumption. In general, test vessels were not aerated during exposure, as we used aerated water to prepare the test concentrations and, furthermore, chironomids can easily tolerate even low oxygen levels [14]. During exposure, mortality and behavior of the larvae (i.e. convulsions correlated with disability to bury themselves into the sediment) were checked and noted every 24 h, and dead animals were removed immediately to avoid contamination of the medium. Larvae were regarded as dead when they were immobile, even after 30 s of mechanical stimulation or whenever they were untraceable. Results were counted as valid when the mean mortality rate in the control was below 10%, which represents the natural mortality rate.

### 2.6. Depletion of nano-Al_2_O_3_

Nanoparticles can change their properties by ageing and biomodification [2, 9, 18]. Consequently, defecated nano-Al_2_O_3_ was sampled to test whether sorption properties of defecated particles differ from pristine ones. Moreover, we aimed at quantifying the uptake of thiacloprid by the larvae. Therefore, after the exposure experiments, larvae were transferred to a single glass Petri dish per treatment (filled with 100 mL filtered and dechlorinated tap water) where they were left to empty their guts for 24 h. No food was added and the dishes were partially wrapped in aluminum foil to reduce light-induced stress [19]. Afterwards, larvae were frozen in liquid nitrogen for chemical analyses. The fecal pellets of animals exposed to 1000 mg/L nano-Al_2_O_3_ as single substance, were sampled with a pipette and transferred into 1.5 mL Eppendorf tubes (Eppendorf, Germany). After collection, fecal samples were centrifuged (14000 rpm, 10 min) and pellets were pooled for sorption experiments.

### 2.7. Sorption measurements

Nano-Al_2_O_3_ was used as a potential sorbent in two forms: pristine and defecated (i.e. present in fecal pellets). Both were suspended in 0.75 ml Millipore water and thiacloprid was added to reach concentrations of 0.3 μg/L or 5.0 μg/L in the supernatant. These concentrations cover the concentration range of the exposure experiments and allow identifying a potential concentration dependence of the thiacloprid sorption coefficient (K_d_-value). Samples were then shaken on an overhead shaker for 15 h and 20 min. After equilibration, thiacloprid concentrations in the aqueous phase were measured in triplicates using a LC-MS method. The liquid chromatography was performed using an Agilent 1260 infinity device including solvent degasser, binary pump, auto-sampler, and column compartment. Thiacloprid was eluted with an isocratic mixture of 50% water and 50% acetonitrile (both acidified with 0.1% formic acid) at 0.6 mL/min for 4 min and detected by tandem mass spectrometry using an Agilent 6490 triple quadrupole mass spectrometer with positive electrospray ionization (ESI+). By this method, the detection limit for thiacloprid was 0.05 μg/L for the experiment with 0.3 μg/L thiacloprid, and, 0.5 μg/L for the experiment with 5.0 μg/L thiacloprid. LC-MS methodological variability was 5-10%.

### 2.8. Chemical analyses

To check whether real thiacloprid concentrations in the experiments corresponded to nominal ones, chemical analyses of the test media were conducted. For each concentration tested, one replicate was analyzed. Water samples were taken from the exposure experiments directly before larvae were introduced, and, additionally, at the end of the exposure. The samples were stored in 2 mL Eppendorf tubes (Eppendorf, Germany) at -18°C. Prior to analysis, samples were centrifuged at 3000 rpm for 3 min, and 1.0 mL supernatant was filtered with a PTFE syringe filter (pore size 0.45 μm, Chromafil^®^ Macherey-Nagel, Germany). Moreover, also the internal thiacloprid concentrations of *C*. *riparius* larvae were measured. Therefore, larvae originating from all treatments, after intestinal voiding, were used. To quantify thiacloprid, a liquid-liquid extraction method was developed based on QuEChERS extraction procedure that is originally used in food analysis and which was recently adjusted to analyze environmental samples and biota [20–22]. For sample extraction and cleanup, 10–30 mg frozen larvae, respectively 10–20 larvae parted in 2–3 replicates, were homogenized in liquid nitrogen and extracted with water and acetonitrile. After a cleanup step containing PSA samples were reconstituted with methanol and filtered with a PTFE syringe filter. Quantification was based on deuterated internal standard thiacloprid-D4 (Sigma Aldrich, USA). Further details can be found in the supporting information S1 Text.

Both the medium and *C*. *riparius* larvae, were analyzed by LC-MS using a QTOF-LCMS. For LC-MS analysis, a 1260 Infinity LC system coupled to a 6550 iFunnel QTOF LC/MS system (Agilent Technologies, Germany) was used. Details on the LC-MS method are also given in the supporting information S1 Text. For water-samples the limit of detection was 0.2 μg/L. Thiacloprid concentrations in larvae were calculated based on peak area of the deuterated internal standard and the detection limit was 1.0 μg/L.

### 2.9. LA-ICP-MS imaging

LA-ICP-MS imaging techniques were used to verify ingestion of nano-Al_2_O_3_ particles by the exposed larvae. This technique was used to display the distribution pattern of aluminum within thin sections of *C*. *riparius* larvae that have been exposed to 1000 mg/L nano-Al_2_O_3_ solely, and from the control treatment. Therefore, after termination of the acute toxicity test, surviving larvae of these treatments were decapitated to facilitate the fixation in 2% glutardialdehyde (Sigma Aldrich, Germany) buffered in 0.005 M cacodylate buffer (sodium cacodylate trihydrate, pH 7.4, Sigma Aldrich, Germany). Samples were stored at least for one week at 4°C before further processing took place. After fixation, samples were de-calcified in 5% TCA (trichloroacetic acid, ≥ 99% p.a., Carl Roth GmbH) in formol (37% p.a., stabilized with methanol, Carl Roth GmbH, Germany), dehydrated with ethanol and routinely processed for Technovit^®^ embedding (Technovit® 7100, Heraeus Kulzer GmbH, Germany). Finally, these samples were sectioned in 7 μm thin sections using a rotary microtome (Leica RM2265, Leica Biosystems, Germany) for LA-ICP-MS imaging. The LA-ICP-MS analysis was performed with a commercially available laser ablation system (LSX-213, CETAC Technologies, USA) using a Nd:YAG laser with a wavelength of 213 nm. The system was coupled to a quadrupole-based ICP-mass spectrometer (iCAP Q, Thermo Fisher Scientific, Germany). The LA parameters relating to spot size, scanning speed, laser energy, and carrier gas flow were optimized based on the best signal-to-noise ratio in combination with highest spatial resolution. The selected samples were completely ablated using a line by line scan (0 μm space) with a laser energy density of 6 J/cm^2^, 20 Hz laser shot frequency, 5 μm spot diameter and 20 μm/s scan speed. The dry aerosol was transported to the ICP-MS system with a carrier gas mixture of helium (0.9 L/min) passing the ablation chamber and argon (0.4 L/min) added downstream the ablation chamber. To compensate drift effects and monitor plasma stability, a gallium (ICP standard, SCP Science, Canada) solution as an internal standard (10 ng/L) was introduced continuously by a PFA (perfluoroalkoxy alkane) nebulizer and a cyclonic spray chamber. For maximum sensitivity and to minimize possible interferences, the measurement was performed in the kinetic energy discrimination mode (KED) with helium as cell gas. The isotopes ^27^Al, ^65^Ga were monitored with dwell times of 0.1 s and 0.05 s, respectively. Gallium ICP-standard (1000 mg/mL) was obtained from SCP Science (Courtaboeuf, France). Nitric acid (67–69%, Optimat) was purchased from Fisher Scientific (Loughborough, UK). The recorded ablation profiles were converted into 2D distribution images and evaluated with the software ImageJ.

### 2.10. Data analysis

Statistical analyses were applied on datasets recorded for all individuals within one treatment (n = 60). To compare the treatments with each other, likelihood ratio tests and, if necessary, Fishers exact test were conducted (α = 0.05) with SAS JMP version 12 (SAS Institute, Germany). To correct for multiple testing, the α-level was adjusted by sequentially Bonferroni correction [23]. Table Curve 2D (Systat Software GmbH, Germany) was used for calculating regression curves and SigmaPlot 13 (Systat Software GmbH, Germany) was used to plot graphs.

### 2.11. Data availability statement

All relevant data are summarized within the paper. Raw data of the experiments are available as Supporting Information S1 Table.

## 3. Results

### 3.1. Sorption measurements

For pristine as well as defecated nano-Al_2_O_3_, 103 ± 5% and 105 ± 5% of the applied 0.3 μg/L thiacloprid were measured to be present in the aqueous phase after equilibration, whereas the recovery rates for 5.0 μg/L thiacloprid were 99 ± 0.6% and 98 ± 1%. Thus, no significant sorption of thiacloprid was found in both types of nano-Al_2_O_3_.

### 3.2. Chemical analyzes

Chemical analyses of water samples from the start and the end of the exposure experiments showed that actual values in solution were, on average, 26.86 ± 9.94% lower than nominal concentrations (Table 1). However, there were only minor deviations among the three treatments including thiacloprid (thiacloprid solely, mixture type A, mixture type B).

**Fig 1:**
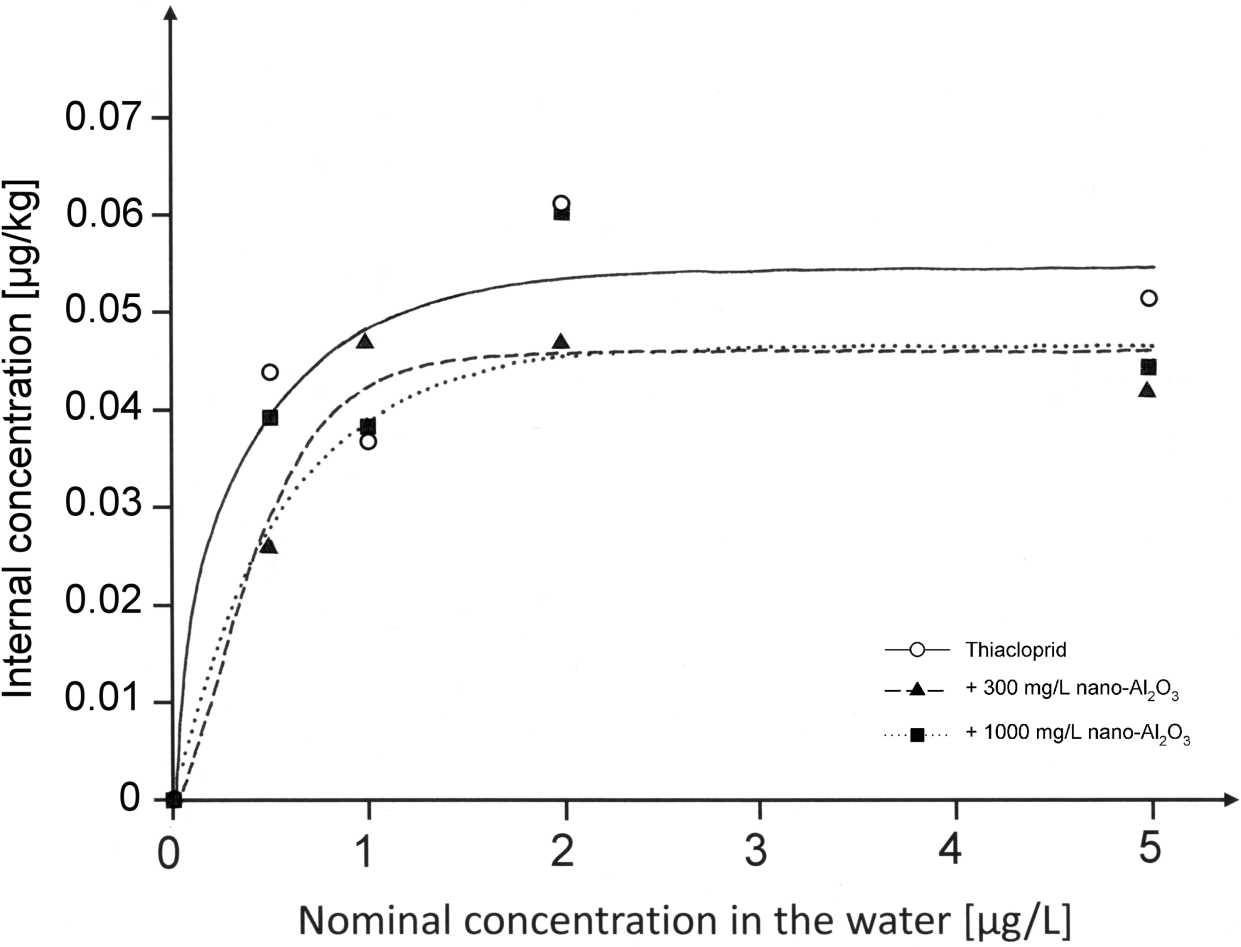
Internal thiacloprid concentrations in *C*. *riparius* larvae [μg/kg] vs nominal concentration in water [μg/L]. Larvae were exposed for 96 h before they were transferred to filtered and dechlorinated tap water for 24 h to empty their guts (n = 1–3). R^2^ of the respective regression curves were 0.91 for Thiacloprid, 0.97 for animals exposed to the mixture including 300 mg/L nano-Al_2_O_3_ and 0.82 for animals exposed to a mixture with 1000 mg/L nano-Al_2_O_3_. Nominal values are shown in this graph, whereas measured concentrations can be obtained from Table 1.

The internal thiacloprid concentrations of chironomids vs. the concentrations in the supernatant are displayed in Fig 1. For all treatments, it was shown that the internal thiacloprid concentrations in the chironomids followed a saturation curve.

Despite general similarity obtained for these regression curves, the exposure to thiacloprid as a single substance showed a (non-significant) tendency to result in a slightly higher saturation level than those exposures that comprised nano-Al_2_O_3_.

### 3.3. Acute toxicity test

**Fig 2:**
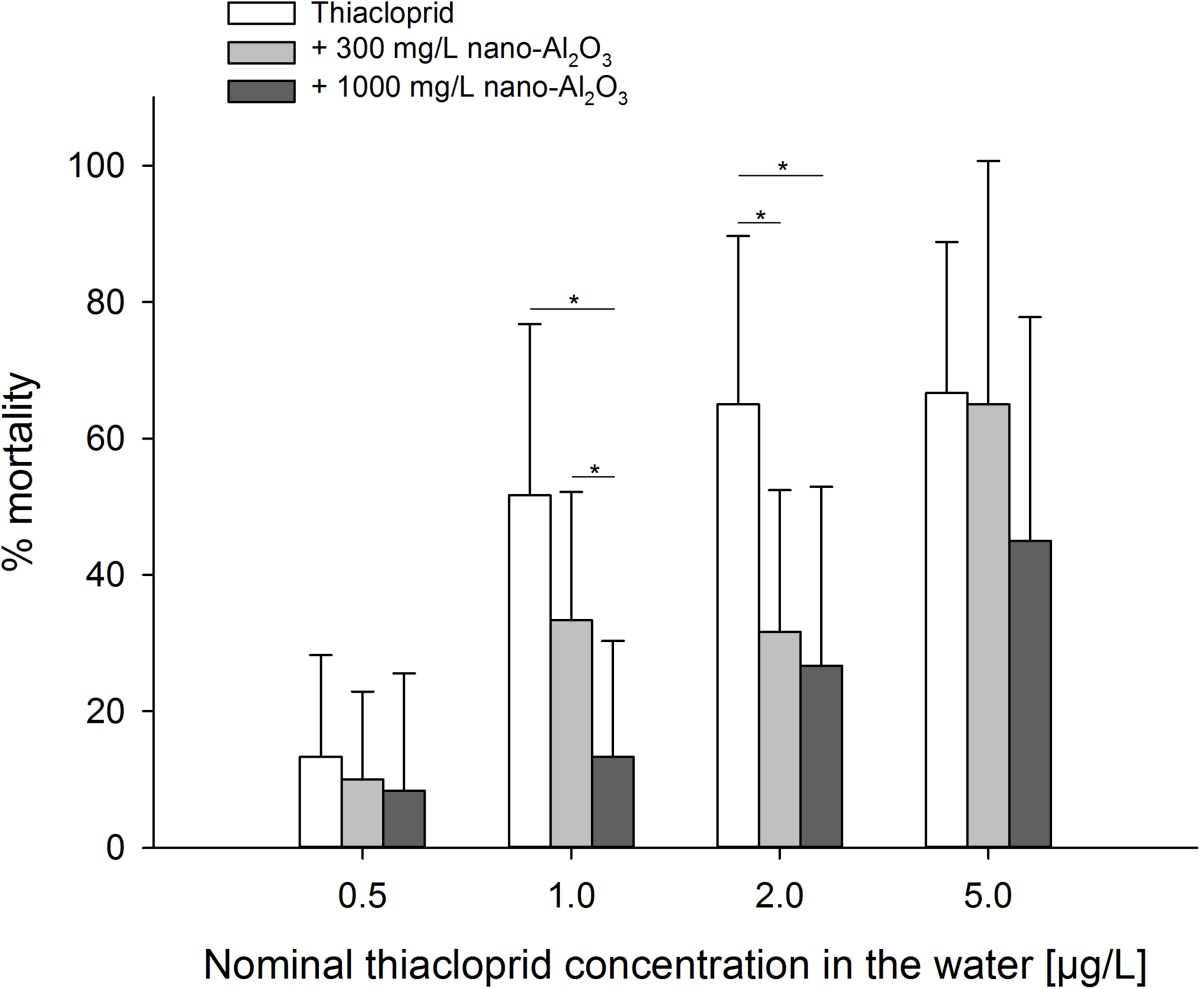
Mortality rates (means ± SD) of larvae per test vessel. Larvae were exposed to either thiacloprid solely, a mixture of thiacloprid and 300 mg/L nano-Al_2_O_3_, or a mixture of 1000 mg/L nano-Al_2_O_3,_ after 96 h of exposure (n = 12). Four comparisons (Likelihood ratio test and Fishers exact test, if necessary) were conducted (one for each thiacloprid concentration) and significant differences between the data displayed at the respective two ends of the horizontal lines are given (* p ≤ α when adjusted according to Holm-Bonferroni’s method [23], Fishers exact test). Nominal values are shown in this graph, whereas measured concentrations can be obtained from Table 1.

The mean mortality rate in the control was 6.5 ± 5.5% after 96 h of exposure thus meeting the criterion of validity (≤ 10% mortality). Concerning the effect of nano-Al_2_O_3_ as single substance, no differences were found between the control and both nano-Al_2_O_3_ concentrations (Likelihood Ratio Test, χ^2^ = 4.182, df = 2, p = 0.1235) after 96 h. In contrast, thiacloprid exerted elevated mortality in a concentration-dependent manner. However, nano-Al_2_O_3_ reduced thiacloprid-induced mortality in the mixture experiments, presumably also in a concentration-dependent manner, since the mean mortality rates of larvae were the lowest when exposed to mixture type A, followed by type B (see Table 1) and thiacloprid solely (Fig 2). However, significant differences were only found at intermediate thiacloprid concentrations.

To be more precise, the addition of 1000 mg/L or 300 mg/L nano-Al_2_O_3_ to 1.0 or 2.0 μg/L thiacloprid resulted in significantly lower mortality rates when compared to 1.0 and 2.0 μg/L thiacloprid solely. The observation that higher concentrations of nano-Al_2_O_3_ result in lower toxicity is furthermore supported by the significant difference between the results obtained for the mixtures type A and type B (see Table 1) when 1.0 μg/L thiacloprid was applied.

**Fig 3:**
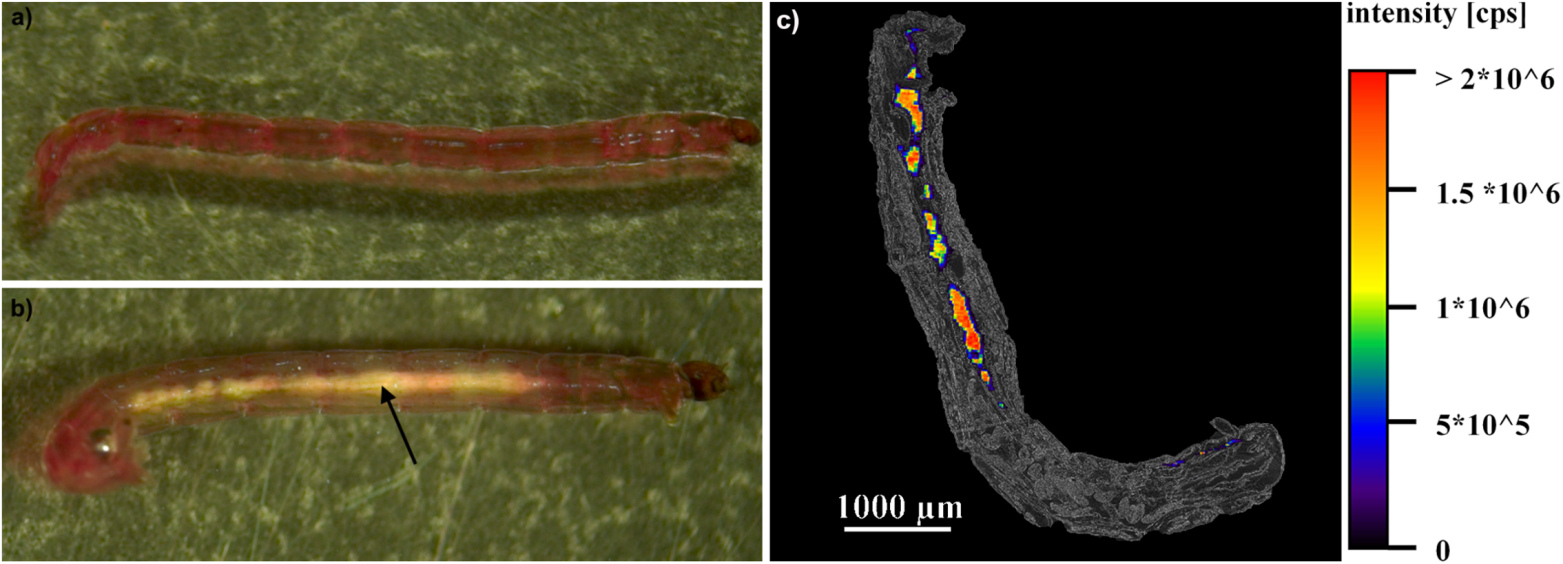
Images of *C*. *riparius* larvae. a) photography of a larva from the control (after 96 h of exposure). b) photography of a larva exposed to 1000 mg/L nano-Al_2_O_3_ (after 96 h of exposure). The arrow marks the agglomeration of nano-Al_2_O_3_ within the gut. c) overlay of an image of a thin section of a larva (after 96 h of exposure to 1000 mg/L nano-Al_2_O_3_) at phase contrast and the aluminum distribution within this section, which was measured by LA-ICP-MS imaging techniques.

Nevertheless, even though the mortality rate of larvae from mixture type A and type B was lower than for animals exposed to thiacloprid solely, it was obvious that all larvae exposed to thiacloprid concentrations > 0.5 μg/L showed strong convulsions during exposure and were unable to bury themselves into the sediment (S2 Fig). This effect was recorded for the vast majority of larvae exposed to 2.0 or 5.0 μg/L thiacloprid after 24 h and for larvae exposed to 1.0 μg/L after 48 h. Moreover, larvae exposed to both nano-Al_2_O_3_ concentrations, solely or in addition to 0.5 μg/L thiacloprid, displayed whitish gut lumina, obviously formed by agglomerations of nano-Al_2_O_3_ (Fig 3B). This phenomenon increased in intensity with particle concentration, as well as with exposure time.

### 3.4. LA-ICP-MS imaging

Aluminum aggregates were detected within the gut lumina of larvae exposed to nano-Al_2_O_3_ (Fig 3C) using LA-ICP-MS. In contrast, no aluminum was detected in individuals from the control treatments. These results were consistent for all analyzed individuals (n = 5).

## 4. Discussion

The aim of the study was to investigate possible interactions of nano-Al_2_O_3_ and thiacloprid that may potentially affect the toxicity of this neonicotinoid insecticide on the non-biting midge *Chironomus riparius*. Due to the nature of this study, we did not focus on environmentally relevant concentrations, but on possible interactions between both substances within an organism. In our experiments, larvae were exposed to both substances solely or in binary mixtures, while mortality and behavior were monitored. The results of our study clearly showed that nano-Al_2_O_3_ particles were non-toxic when applied solely, but prevent (or at least delayed) the negative effects of thiacloprid in mixture experiments, even though we could not measure any sorption of thiacloprid to the nanosized particles.

Our first result, the lack of toxic effects of nano-Al_2_O_3_, diverges from a former study of Oberholster et al. [24], who tested the effect of nano-sized α-alumina and γ-alumina (both with an average size range < 100 nm) on the survival rate of 2^nd^– 3^rd^ instar larvae of *Chironomus tentans* and observed a significant decrease in survival of the larvae compared to the control. Also other parameters like growth length, DNA strand breaks, or catalase enzyme activity indicated harmful effects of α-alumina and γ-alumina [24]. The presence of toxic effects in the study of Oberholster et al. [24] and in other studies (e.g. [25, 26]) contradicts with the absence of effects in our experiments, but can be attributed to differences in endpoints, test setups, test species and life stages. In this context, it must be noted that the transferability of results between different nanoparticle studies must be handled with extreme care, since nanoparticles – even if they are chemically identical–dramatically change their properties and, likely also their ecotoxicity (or mode of action) when characteristics like size or shape differ [1, 9, 24, 27, 28]. In our case, the absence of toxicity is likely caused by the relatively large size of the nano-Al_2_O_3_ particles, since larger particles tend to have less toxic impacts than smaller ones of the same chemical composition [6, 24, 26]. Next to the result that the nano-Al_2_O_3_ did not affect the mortality rate of fourth instar larvae of *C*. *riparius*, we observed that the mortality rates increased in all treatments with increasing thiacloprid concentration. This trend was expected, since former studies already have revealed the strong toxicity of thiacloprid on the non-target organism *C*. *riparius* [13]. Moreover, larvae exposed to higher concentrations than 0.5 μg/L thiacloprid showed heavy convulsions and were unable to bury themselves in the sediment. These convulsions, and also mortality, were likely caused by a permanent stimulus of the nervous system by thiacloprid, which acts as an agonist of the nicotinic acetylcholine receptor [29]. Behavioral impairments of *C*. *riparius* larvae exposed to thiacloprid or imidacloprid have also been found by Langer-Jaesrich et al. [13] and Azevedo-Pereira et al. [30, 31]. However, effect concentrations of thiacloprid were much higher in our study compared to the literature. For example, Langer-Jaesrich et al. [13] report LC_50_ values of 5.18 or 1.5 μg/L thiacloprid, when larvae were exposed for 10 or 17 days, respectively. We assume that the lack of food adds stress to the larvae, which may have enhanced the toxicity of thiacloprid synergistically. In controls, however, none of the investigated endpoints was affected by starvation.

Although toxic effects of thiacloprid were demonstrated, we observed that the mortality rate of the midge larvae was significantly reduced in the mixture treatments with intermediate thiacloprid concentrations, compared to thiacloprid as single substance. Consequently, the presence of nano-Al_2_O_3_ had a positive effect on the survivability, which was also found to be concentration dependent. However, this effect was absent for the lowest and highest thiacloprid concentrations, probably since the concentrations of the insecticide were either too low to cause any effect, or too high in cases when the protective effect of nano-Al_2_O_3_ particles could not compensate the neurotoxic effects of thiacloprid. Although it is already known that nanoparticles, particularly those which are present in the intestinal tract, can reduce negative impacts of toxins [2, 6], the underlying mechanisms are scarcely described until now. However, as the rationale for our study was to test interactions that are not based on adsorption, we explicitly had chosen particles with suitable properties, which were successfully confirmed for pristine and defecated nano-Al_2_O_3_ particles by sorption experiments. So, we can definitely exclude reduced bioavailability of thiacloprid to be caused by sorption processes both outside or inside the animals. Thus, the reduced toxicity is likely a result of another toxicological mechanism. From our point of view, it is more likely that nano-Al_2_O_3_ either changes the physico-chemical properties of the intestinal tract (e.g. the pH), or it forms a physical barrier within the intestinal tract, i.e. by covering the gut epithelium or by thickening the peritrophic membrane. The latter assumption is based on the observation that nano-Al_2_O_3_ was ingested by the larvae and aggregated within the gut. Such ingestion and aggregation or accumulation of nano-sized Al_2_O_3_ have already been described by Zhu et al. [26] for *Daphnia magna*, in which also other nanoparticles, e.g. nano-C_60_ [11], carbon nanotubes [32] or TiO_2_ [33] aggregated/accumulated. For chironomids, the ingestion of nano-Al_2_O_3_ was obviously oral, since the larvae are deposit-feeders and nano-Al_2_O_3_ particles tend to settle down on the substrate [14]. We assume that the feeding behavior of, and, consequently, the uptake of nano-Al_2_O_3_ particles by larvae exposed to higher thiacloprid concentrations (> 0.5 μg/L) was impaired by the insecticide. Thus, in these individuals, no aggregates of nano-Al_2_O_3_ that are visible with the naked eye could be detected. Since we have demonstrated a protective effect of nano-Al_2_O_3_ also for individuals exposed to higher thiacloprid concentrations, however, we assume that they ingested nano-Al_2_O_3_ as well, but to a lower extent. Moreover, it is likely that most of the nano-Al_2_O_3_ was ingested within the first few hours of the exposure experiments, before thiacloprid lead to convulsions and impaired the foraging behavior. This might also explain the positive correlation between survival and nano-Al_2_O_3_ concentration. We expect that larvae, exposed to high concentrations of nano-Al_2_O_3_, ingested more of these particles which, at least in the short term, led to benefits during further exposure, e.g. by enhancing the physical barrier within the gut. Moreover, the mortality rates and the observed protective effect of nano-Al_2_O_3_ are reflected in the regression curves calculated for the internal thiacloprid concentrations in the midge larvae. These curves showed - although the internal concentrations were rather similar - a trend towards a higher saturation level when thiacloprid was administered as a single substance compared to larvae that have been exposed to mixtures. Furthermore, the data revealed thiacloprid to accumulate in the larvae despite of its rather low log K_OW_ of 1.26. However, it cannot be excluded that a time span of 24 h might be too short for the larvae to excrete substantial amounts of ingested thiacloprid and/or its metabolites. This assumption is also supported by a study of Azevedo-Pereira et al. [31], which showed that *C*. *riparius* larvae exposed to a neonicotinoid for 96 h recovered only after 6 days in clean medium.

Given that the animals suffer from thiacloprid even in the mixture experiments, we expect that the nano-Al_2_O_3_ just deferred their death and did not totally prevent it. In addition, the aggregation of nano-Al_2_O_3_ within the intestinal tract of organisms may lead to decreased uptake rates for various other, also beneficial substances, not only thiacloprid. Under such conditions, the food ingestion and nutrient absorption in these animals are reasonably suspected to be affected [33]. All this probably does not affect the mortality rate of *C*. *riparius* larvae under acute treatment, but will likely impair the animals and possibly even increase mortality under chronic conditions. At the moment, however, we can only speculate about the chronic effects of nano-Al_2_O_3_ and, therefore, further experiments with extended exposure times, e.g. experiments following the OECD guidelines 218/219 [15, 16], should be conducted. These results will help to interpret the results of future nanoparticle studies, since an obstruction of the gut of sediment or particle-feeders by an aggregation/accumulation of nanomaterials might be of importance for the risk assessment of these substances. Nevertheless, our present results on acute effects already revealed a new and previously undetected kind of interaction between nanoparticles and pollutants within an organism. This observation highlights the need of more research about nanomaterials, particularly in view on their interactions with other chemicals in ecotoxicology.

## Supporting information

S1 Fig Transmission electron microscopy (TEM) image of nano-Al_2_O_3_ particles. The sample was prepared by dispersing the powdery material in ethanol; one drop of the suspension was dried on the copper grid with a holy carbon film.
(TIF)

S2 Fig Percentages of living *C*. *riparius* larvae showing convulsions during exposure (96 h). Values are shown for different nominal concentrations of thiacloprid in water (a-d).
(TIF)

S1 Text Detailed description of chemical analyses. (DOCX)

S1 Table Data set. (XLSX)
