# Correction: Nano-sized Al_2_O_3_ reduces acute toxic effects of thiacloprid on the non-biting midge *Chironomus riparius*

**DOI:** 10.1371/journal.pone.0179786

**Published:** 2017-06-12

**Authors:** Carla S. Lorenz, Anna-J. Wicht, Leyla Guluzada, Leilei Luo, Leonie Jäger, Barbara Crone, Uwe Karst, Rita Triebskorn, Yucang Liang, Reiner Anwander, Stefan B. Haderlein, Carolin Huhn, Heinz-R. Köhler

In Fig 1, the y-axis is labeled incorrectly. Please see the correct Fig 1 here.

**Fig 1:**
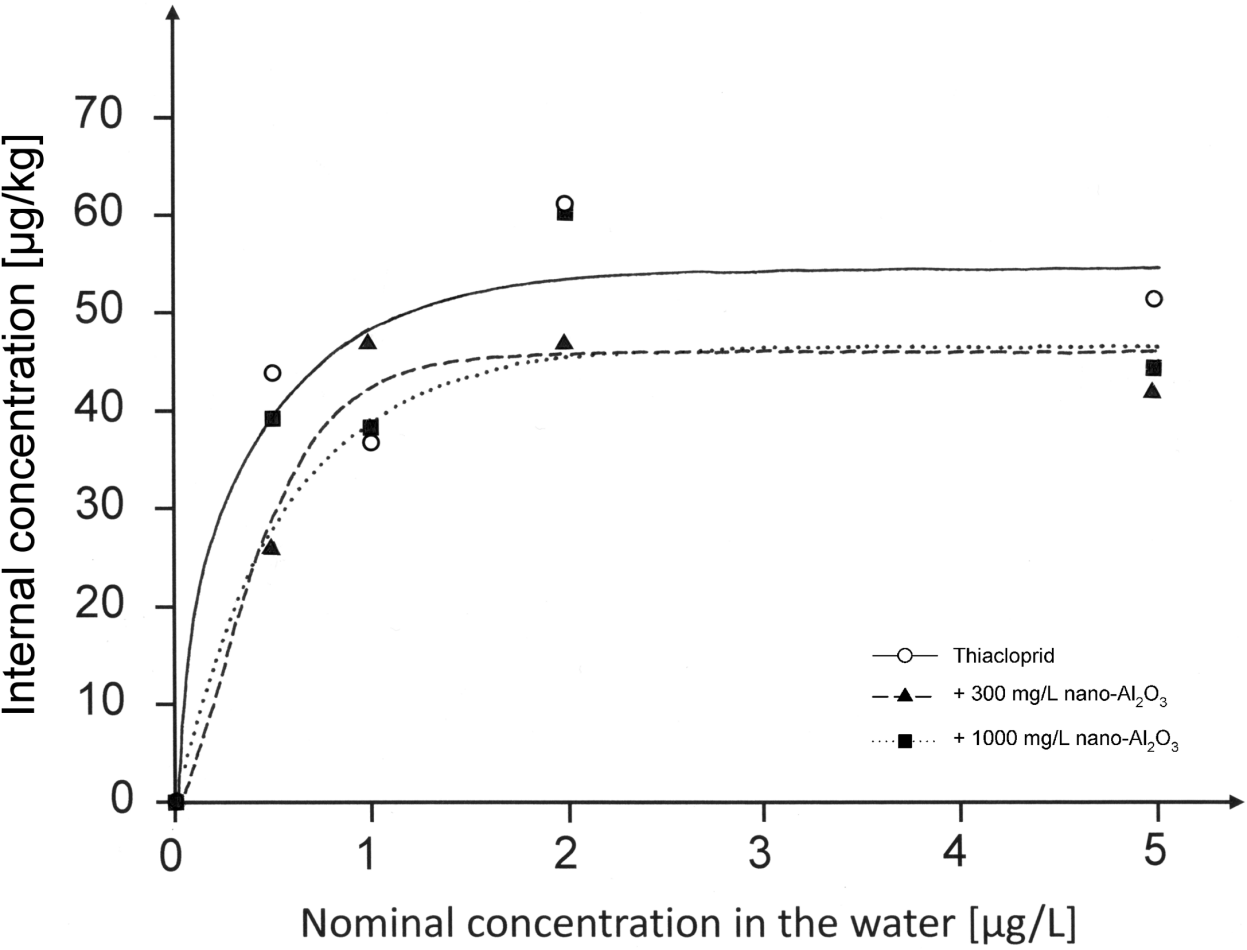
Internal thiacloprid concentrations in *C*. *riparius* larvae [μg/kg] vs nominal concentration in water [μg/L]. Larvae were exposed for 96 h before they were transferred to filtered and dechlorinated tap water for 24 h to empty their guts (n = 1–3). R^2^ of the respective regression curves were 0.91 for Thiacloprid, 0.97 for animals exposed to the mixture including 300 mg/L nano-Al_2_O_3_ and 0.82 for animals exposed to a mixture with 1000 mg/L nano-Al_2_O_3_. Nominal values are shown in this graph, whereas measured concentrations can be obtained from Table 1.
