# Supplementary figures and images for: Nano-sized Al2O3 reduces acute toxic effects of thiacloprid on the non-biting midge Chironomus riparius
Source: PLoS One. 2017 May 2;12(5):e0176356. doi: 10.1371/journal.pone.0176356 (PMC5413047; doi:10.1371/journal.pone.0176356)

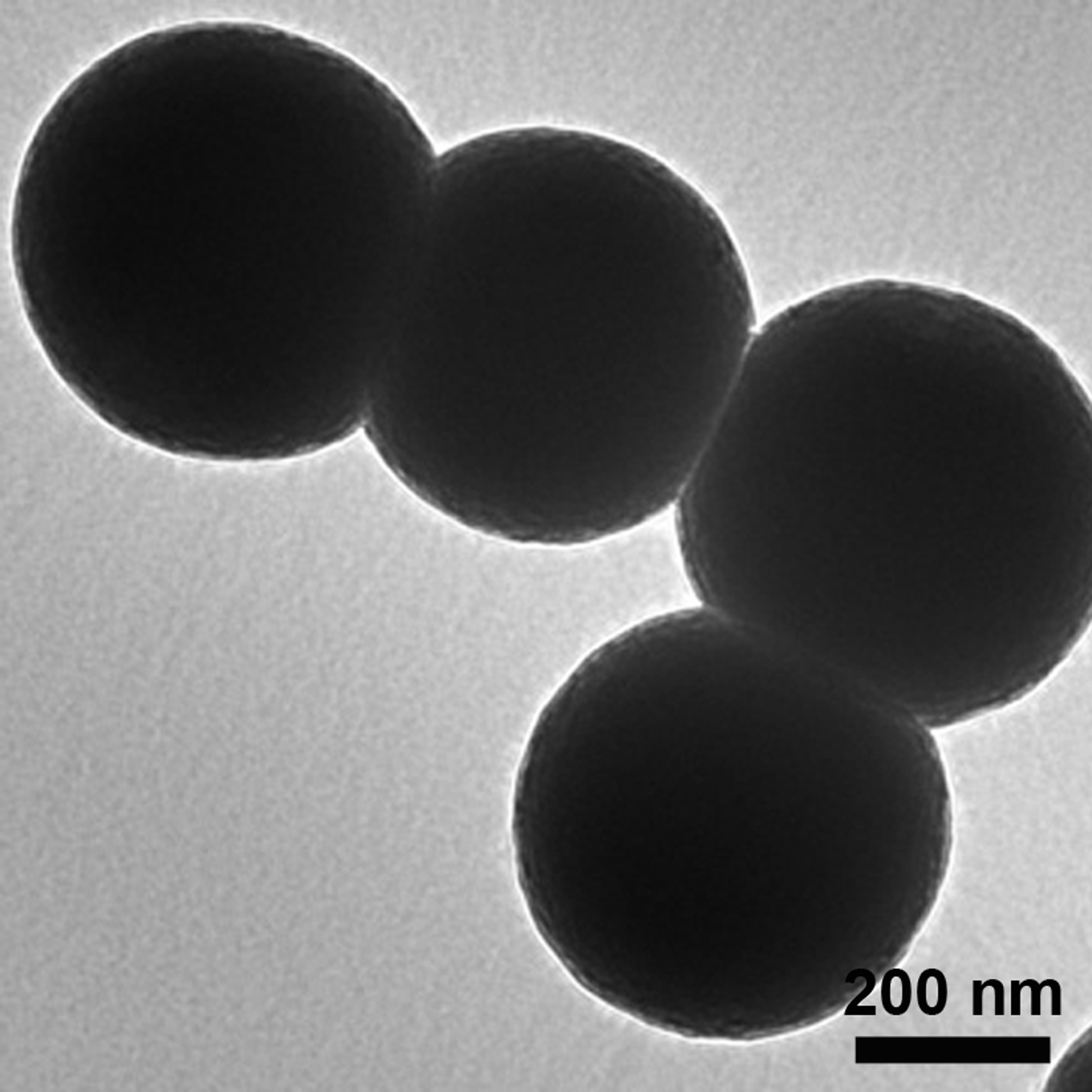

Supplement: S1 Fig — The sample was prepared by dispersing the powdery material in ethanol; one drop of the suspension was dried on the copper grid with a holy carbon film. (TIF) [file pone.0176356.s001.tif]

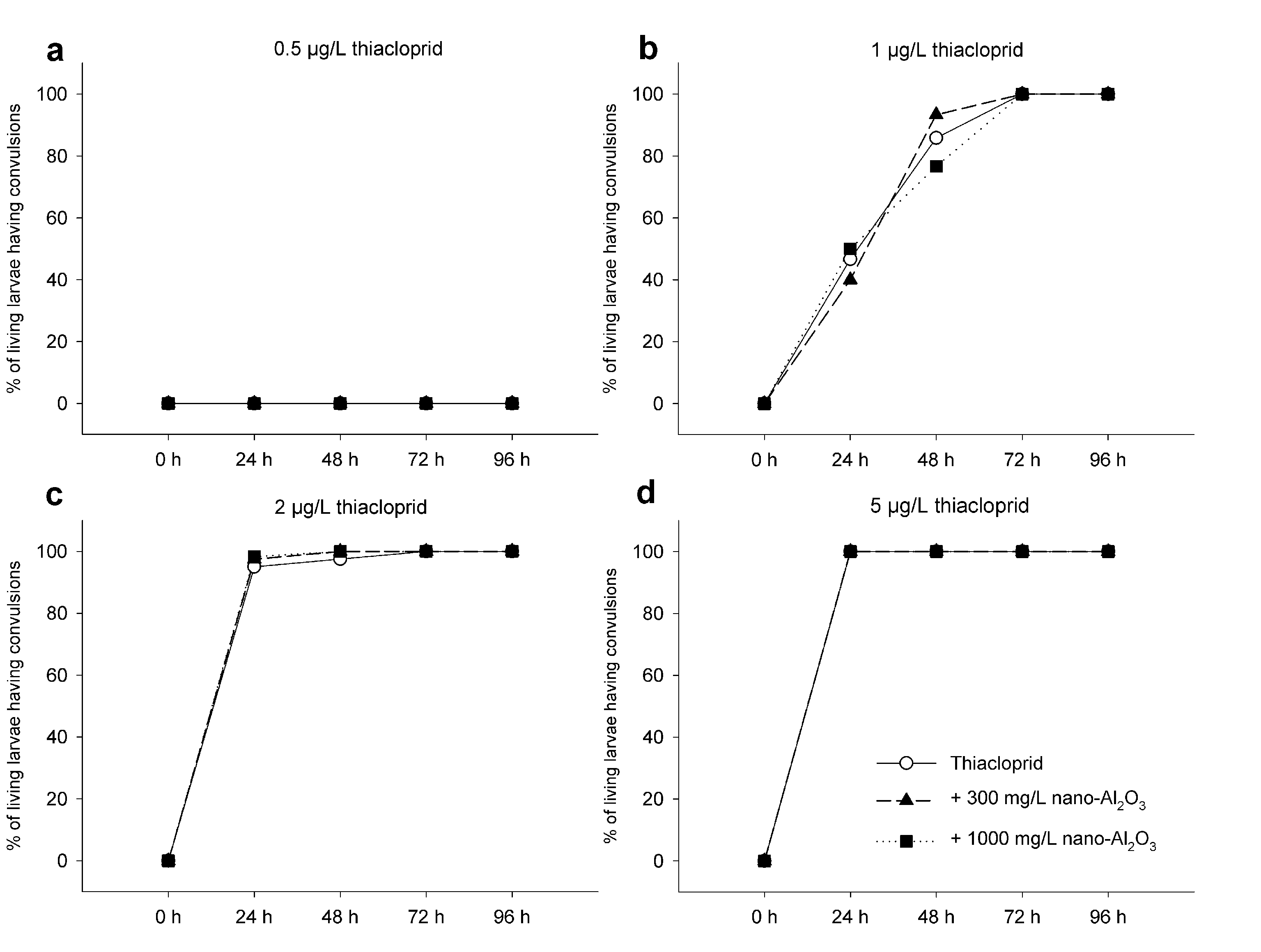

Supplement: S2 Fig — Values are shown for different nominal concentrations of thiacloprid in water (a-d). (TIF) [file pone.0176356.s002.TIF]
