# Supplementary material for: Nano-sized Al2O3 reduces acute toxic effects of thiacloprid on the non-biting midge Chironomus riparius
Source: PLoS One. 2017 May 2;12(5):e0176356. doi: 10.1371/journal.pone.0176356 (PMC5413047; doi:10.1371/journal.pone.0176356)
Supplement: S1 Text — (DOCX) [file pone.0176356.s003.docx]

**Supplementary material**

**S1 Text: Detailed description of chemical analyses**

We used a modified QuEChERS extraction procedure for the quantification of thiacloprid. Therefore, we homogenized 10-30 mg frozen in liquid nitrogen with a Micro-homogenizer (Carl Roth GmbH, Germany) in a 2.5 mL tube (Eppendorf, Germany) larvae for sample extraction and cleanup. 20 µL deuterated internal standard thiacloprid-D4 (Sigma-Aldrich, USA) were added, resulting in a final concentration of 8.0 µg/L in the analyzed sample. A volume of 0.5 mL water (Chromasolv^®^, Sigma-Aldrich, USA) and 0.5 mL acetonitrile were added, and the sample was shaken for 20 s using a Vortex mixer. After addition of 25 mg NaCl (Sigma-Aldrich, USA) and 75 mg anhydrous MgSO_4_ (Sigma-Aldrich, USA) the sample was immediately shaken for 20 s using a Vortex mixer and centrifuged for 2 min at 10000 rpm. A volume of 0.5 mL of the acetonitrile phase were transferred to 12 mg primary-secondary amine (PSA, Agilent technologies, Germany) and 90 mg anhydrous MgSO_4_, then shaken for 20 s and centrifuged for 2 min at 10000 rpm. The supernatant was concentrated in a nitrogen stream at room temperature, and the residue was reconstituted in 250 µL methanol and filtered with a PTFE syringe filter (pore size 0.45 µm, Chromafil^®^, Macherey-Nagel, Germany).

For the LC-MS analyses we injected aliquots of 10 µL onto a Zorbax Eclipse Plus C18 column (2.1x150 mm, 3.5-Micron, narrow bore, Agilent Technologies, USA) with a narrow bore guard column (2.1 x 12.5 mm, 5-Micron, Agilent Technologies). A jetstream electrospray ionization (ESI) source was operated with a nebulizer pressure of 35 psig, drying gas temperature of 160 °C, a flow rate of 16 L/min, and a fragmentor voltage of 360 V. In the positive mode, capillary voltage was set to -4000 V, skimmer voltage to 65 V and the nozzle voltage to -500 V. The mass range was 80 - 1200 m/z with a data acquisition rate of 1 spectrum/s. The sheath gas temperature was set to 325 °C with a flow rate of 11 L/min. For internal calibration, purine and HP0921 (Agilent Technologies, USA, m/z = 121.0508, 922.0097) were used and the mass range was set to 70 ppm. A gradient elution was performed at a flow rate of 0.3 mL/min using water containing 0.1 % formic acid and methanol. The initial content of 95 % water was decreased after 1 min to 5 % water over 7 min and, after another 7 min at 5 %, increased to 95 % water over 0.5 min.

Data analysis was performed with MassHunter software (Agilent Technologies, USA). For quantification of water-samples, a calibration curve in the range of 0.2-10 µg/L thiacloprid was used (r^2^= 0.9991) and the limit of detection was 0.2 µg/L. Thiacloprid concentrations in larvae were calculated based on peak area of the deuterated internal standard of 8.0 µg/L and the detection limit was 1.0 µg/L.
